# CTLA-4 and PD-1 Control of T-Cell Motility and Migration: Implications for Tumor Immunotherapy

**DOI:** 10.3389/fimmu.2018.02737

**Published:** 2018-11-27

**Authors:** Monika C. Brunner-Weinzierl, Christopher E. Rudd

**Affiliations:** ^1^Department of Experimental Pediatrics, University Hospital, Health Campus Immunology, Infectiology and Inflammation, Otto-von-Guericke-University, Magdeburg, Germany; ^2^Research Center-Maisonneuve-Rosemont Hospital (CRHMR), Montreal, QC, Canada; ^3^Département de Medicine, Université de Montréal, Montreal, QC, Canada

**Keywords:** CTLA4, check-point blockade, cancer, T-cell, motility, migration, PD1, immune surveillance

## Abstract

CTLA-4 is a co-receptor on T-cells that controls peripheral tolerance and the development of autoimmunity. Immune check-point blockade (ICB) uses monoclonal antibodies (MAbs) to block the binding of inhibitory receptors (IRs) to their natural ligands. A humanized antibody to CTLA-4 was first approved clinically followed by the use of antibody blockade against PD-1 and its ligand PD-L1. Effective anti-tumor immunity requires the activation of tumor-specific effector T-cells, the blockade of regulatory cells and the migration of T-cells into the tumor. Here, we review data implicating CTLA-4 and PD-1 in the motility of T-cells with a specific reference to the potential exploitation of these pathways for more effective tumor infiltration and eradication.

## Introduction

**Figure 1:**
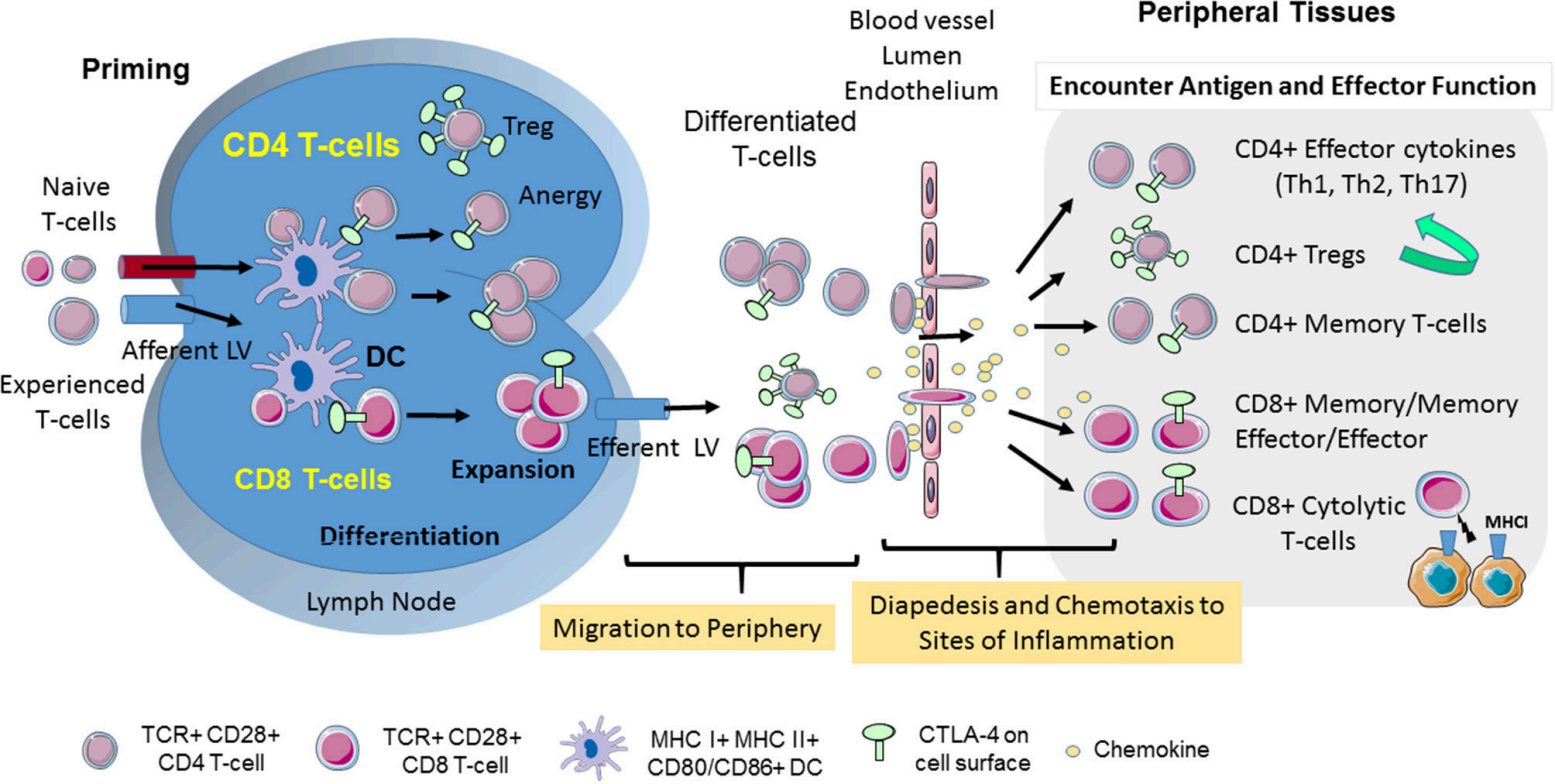
CD28 and CTLA-4-mediated T-cell motility. T-cell response is initiated in secondary lymphoid organs. Naïve and experienced T-cells enter lymph nodes where they encounter antigen presented by DCs. CTLA-4 limits the interaction of CD4+ T-cells with DCs in the reverse-stop signal model involving an increase in T-cell motility, and a raising of the threshold needed to activate T-cells. In the “reverse-stop signal model”, CTLA-4 induces T-cell motility and limits T-cell binding to DCs during antigen-presentation (1, 2). Reverse stop-signaling might also promote the egress of T-cells as mediated by responses to Sphingosine-1-phosphate (S1P) and chemokines. T-cells then migrate from the vasculature to infected tissue via a combination of chemokines and CTLA-4. CTLA-4 can alter motility by up-regulating key chemokine receptors CCR5 and CCR7 and the sensitivity toward the chemokines (3, 4). In the presence of antibody blockade, T-cells accumulate in the blood and remain circulating in the body (3). Upon entry into tissues, different T-cell subsets play important roles in determining the immune response to infection. The scheme was drawn using pictures from Servier Medical Art.

**Figure 2:**
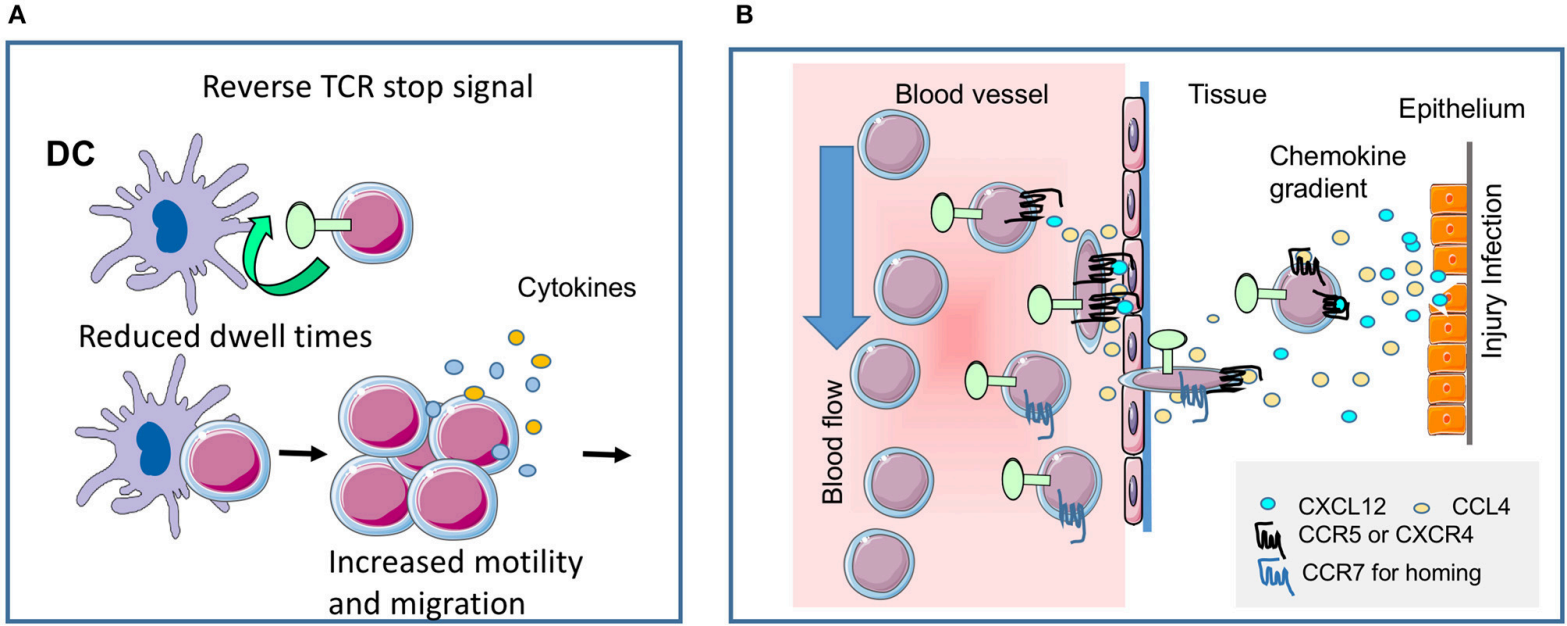
CTLA-4 regulates T-cell motility. **(A)** Reverse-stop signal model of CTLA-4 (and PD-1). CTLA-4 induces T-cell motility and limits T-cell binding to DCs during antigen-presentation (1, 2). Agonistic CTLA-4 ligation could directly activate the motility of T-cells and thereby interfere with the dwell times of cells with DCs presenting antigenic peptide. PD-1 can function in a similar way (5). **(B)** CTLA-4 modulates response to chemokines. Chemokine gradients attract T-cells to the site of injury and inflammation. CTLA-4 can alter motility by up-regulating key chemokine receptors CCR5 and CCR7 and the sensitivity toward the chemokines CCL4 (MIP-1β), CXCL12 (SDF1α) and CCL19, but not CXCL9 (MIG) (3). The scheme was drawn using pictures from Servier Medical Art.

T-cells circulate continuously between blood, lymphoid tissues and lymph nodes as a mechanism to encounter and respond to foreign antigen. The movement or motility of T-cells involves integrin and selectin mediated adhesion, increased velocity and arrest, chemotaxis to sites of inflammation, homing back to compartments of initial antigen contact, transmigration to enter tissues and movement inside tissues (Figure 1). Antigen-experienced T-cells extravase into non-lymphoid tissue and travel back via lymphatic vessels. In other instances, i.e., in the lymph nodes where foreign antigen is presented to T-cells by dendritic cells (DCs), integrins such as lymphocyte function-associated antigen 1 (LFA-1) are activated by chemokines and antigen-receptor (T-cell receptor; TCR) ligation to bind to their ligands inter-adhesion molecules (ICAMs) to facilitate the “stop signal” for T-cell-dendritic cell (DC) conjugate formation (Figures 1, 2A). The operations of adhesion and chemokine reactivity from blood to tissue involves multi-step transmigration (6).

Integrin-activation supports activation of chemokine receptors that directs migration of T-cells from blood into tissues or back home into lymph nodes and spleen. The movement of T-cells responds to intrinsic and environmental clues. Chemokines play central roles in inducing the movement of mammalian cells to various niches of the immune system (7, 8). Chemokines effect the motility of CD4 and CD8 T-cells, as well as, suppressor regulatory T-cells (Tregs), although not always in a similar fashion (9, 10) (Figure 1). T-cells in distinct differentiation states such as naïve, effector, or memory T-cells move differently in the same environment to the same clues. Classically, the presence of sensitive CCR7 mediates homing of T-cells to lymph nodes and spleen, while the presence of CXCR5 in follicular T-cells dictates their movement to germinal centers, whereas CXCR3 and CCR5 directs them to the site of injury and inflammation (11). Antigen-experienced T-cells involve movement over long distances were infection might occur, while naïve cells tend to explore the local environment over shorter distances in search of presented antigen (12). Co-receptors such as CD28 and CTLA-4 also modulate these pathways for effective migration.

## CD28

CD28 plays a central role in providing a second signal needed for T-cell activation (13, 14). Activation signals from the antigen-receptor (TCR) are modified by signals from CD28 and other co-receptors (15–17). CD28 signals via the binding of the lipid kinase phosphatidylinositol- 3-kinase (PI3K) and the adaptor GrB2-SOS complex (18, 19) and p56^lck^ which recruits the protein kinase C (20). It changes the organization of the cytoskeleton (16, 21, 22) and promotes the localization of T-cells to target tissue following antigenic priming (23). With this, it promotes egress from lymphoid tissue and migration to sites of inflammation. Although the downstream pathways that link CD28 to adhesion and migration are not fully understood, loss of CD28 binding to PI3K changes localization to tissues and may favor primed T-cell migration to non-lymphoid tissues (24).

## CTLA-4

Check-point blockade of cytotoxic T lymphocyte antigen 4 (CTLA-4, CD152) is a major focus in tumor immunotherapy (25, 26). Ipilimumab, a humanized antibody against the inhibitory co-receptor CTLA-4, was the first checkpoint-block mAb to be approved (27). It is thought to act during neo-antigen presentation in lymph nodes and can affect primary and secondary responses to antigen. The loss of CTLA-4 in mice leads to a dramatic lymphoproliferative disorder where animals die within 3–5 weeks of age. Activated CD4 T-cells show an increased localization and infiltration of non-lymphoid and lymphoid organs where they accumulate in lymph nodes, the heart, liver, and pancreas (28–30). Other *in vivo* models involving antigen-specific T-cell responses combined with CTLA-4 blockade using specific antibodies (31, 32) or reduced CTLA-4 expression (10) support the notion that CTLA-4 can control T-cell infiltration into allo-grafts and tumors.

CTLA-4 dampens T-cell responses via cell intrinsic and extrinsic pathways. Intrinsic events include the inhibition of protein translation, recruitment of phosphatases, activation of ubiquitin ligases, inhibition of cytokine receptor signaling (33–38) and inhibition of lipid microdomain formation on the surface of T-cells (39). CTLA-4 has also been reported to bind to the phosphatases SHP2 and PP2A (34, 40, 41), although the cytoplasmic tail lacks ITIMs for SHP2 binding (42) and PP2A also binds to CD28 (34). Cell extrinsic events include the competition for CD28 in binding to its ligands CD80/86 (43), the removal of CD80/86 (44), the release of suppressive indoleamine (2,3)-dioxygenase (IDO) and the modulation of Treg function (35, 45). Each model has strengths and weaknesses. While competition with CD28 can occur, the induction of autoimmune disease in *Ctla-4*^−/−^ mice depends on a C-terminal intracellular proline CD28 motif in *in vivo* co-stimulation (46). Similarly, while CD80/86 can be trans-endocytosed from the surface of DCs by CTLA-4 (44), the level of CD80/86 removal *in vivo* is low and the ligands can be rapidly re-expressed on presenting cells. Further, whereas the selective deletion of CTLA-4 on FoxP3^+^ Tregs can delay the onset of disease, mice still die within 2–3 months (35, 45). Moreover, the CTLA-4 YVKM motif binding to PI3K activates pro-survival signals (47, 48) and LFA-1 adhesion (49). Beyond this, the TCR/CD3 mediated stop-signal is decoupled in T-cells from CTLA-4 deficient mice (50) and CTLA-4 has regulatory effects on homeostasis which modulates overall levels of peripheral T-cells (35). It is likely that multiple factors account for the auto-proliferative phenotype in the *Ctla-4*^−/−^ mice.

## PD-1

PD-1 is a member of the CD28 superfamily which negatively regulates T-cell activation. Blockade of the inhibitory co-receptor PD-1 or its ligand ligand PD-L1 has shown survival rates of 20–30% in treating various types of cancer (27, 51). Negative signals are generated by a cytoplasmic immunoreceptor tyrosine-based switch motif (ITSM) motif that binds to the protein tyrosine phosphatase SHP-2 and which can limit B-cell and T-cell signaling (52, 53). While PD-1-SHP-2 inhibits TCR and/or CD28 signaling (52–54), it is unclear whether PD-1 signals in the same manner in different T-cell subsets. To date, PD-1 has been found to primarily regulate the cytolytic effector function of CD8^+^ cells (55, 56). Anti-PD-1 immunotherapy also depends on the expression of CD28 (57).

## CTLA-4 and PD-1 regulation of T-cell motility

The massive infiltration of organs observed in the *Ctla-4*^−/−^ provided the first clue that the co-receptor could alter migration of T-cells. Whether this was due to the hyper-activated state of activated *Ctla-4*^−/−^ CD4 T-cells and/or was related to a direct effect of the co-receptor on mechanisms that affected T-cell motility and/or migration was unclear. An initial clue suggesting that a cell intrinsic pathway might be induced by CTLA-4 was apparent in the observation that T-cells in *Ctla-4*^−/−^mice expressing a tailless form of the gene showed alterations in cell migration (58). Further, the acceleration of allograft rejection by CTLA-4 blockade *in vivo* is associated with more severe mononuclear cell infiltration (59). In addition, depletion of CTLA-4 on T-cell subpopulations *in vivo* showed that while CTLA-4 on Tregs inhibits the aberrant activation of T-cells, the expression of CTLA-4 on conventional T-cells prevents aberrantly activated T-cells from infiltrating and fatally damaging non-lymphoid tissues (60).

CTLA-4 has been shown to engage mechanisms linked to T-cell movement (1–4, 61) (Figures 1, 2). It was first shown to activate LFA-1 adhesion via increased clustering of integrin receptors (49). YVKM motif binding to PI3K mediates this adhesion (49). This observation suggested that distinct motifs in co-receptor might mediate different intracellular events. Further, it offered the interesting possibility that CTLA-4 could generate both negative and positive signals. Indeed, a precedent was seen in nerve growth factor (NGF) signaling where the binding of PI3K determined whether positive or negative signals leading to apoptosis or cell death were generated (62). The absence of PI3K binding resulted in proapoptotic signaling via the receptor.

One key function of CTLA-4 is to interfere with the ability of T-cells to form stable conjugates with antigen-presenting cells (APCs) (Figure 2A). In the “reverse-stop signal model”, CTLA-4 was found to induce T-cell motility and to limit T-cell binding to DCs during antigen-presentation (1, 2). CTLA-4 ligation with specific antibodies activates the motility of T-cells, while CTLA-4 on T-cells interferes with the dwell times of cells with DCs presenting antigenic peptide. Strikingly, antigen-specific *Ctla-4*^−/−^ T-cells continue to move even in the presence of antigen (1). Similarly, the expression of CTLA-4 in transformed cell line, Jurkat promotes its motility (63). In terms of cell biology, CTLA-4 ligation induces a polarized morphology typical of motile T-cells, which in turn depends on the mediator's phosphatidylinositol 3-kinase, Vav-1, Cdc42, and myosin light chain kinase (64). From this, we proposed that the ability of CTLA-4 to limit contact times reduced the efficacy of TCR ligation and signaling which in turn raises the threshold needed to activate T-cells (2). Antigen-attracted T-cells competent for CTLA-4 move specifically to sites of inflammation and easily home to lymph nodes *in vitro* and *in vivo*, whereas CTLA-4 incompetent T-cells migrate to a lesser extent (3, 60).

It is noteworthy that the effects of CTLA-4 on motility may not operate equally in all T-cells. The reverse-stop effects appear limited to conventional T-cells (Tconvs) (9). It does not operate as efficiently in regulatory T-cells (9), or in anergic T-cells (5). Further, in certain antigen-presentation systems, the blockade of CD80/CD86 itself was as effective as CTLA-4 blockade in promoting the dissociation of T-cells from DCs and increased motility (65). While blockade of CD80/86 will also affect the induction of activation signals from CD28, and indirectly act to terminate T-cell-APC binding, it is also possible that the steric blockade of CTLA-4 with CD80/86 might release T-cells in a manner seen with reverse-stop signaling. Lastly, we also observed that T-cells from *Ctla-4*^−/−^ mice are unable to arrest when ligated with anti-CD3 (50). The reason for this is unclear but may involve the heightened activation status of T-cells in an inflamed immune environment. It provides a potential explanation for the massive infiltration of all organs of the *Ctla-4*^−/−^ mice with T-cells. Conversely, the expression of CTLA-4 on conventional T-cells prevents aberrantly activated T-cells from infiltrating and fatally damaging non-lymphoid tissues (60).

In a second pathway of regulation, CTLA-4 can alter motility by up-regulating key chemokine receptors CCR5 and CCR7 and increasing their sensitivity to chemokines CCL4 (MIP-1β), CXCL12 (SDF1α) and CCL19, but not CXCL9 (MIG) (3) (Figure 1, middle; Figure 2B). We have proposed a model for chemotaxis that integrates CD28 and CTLA-4 signals via the G protein-coupled receptor kinase GRK that its phosphorylation of chemokine receptors for de-sensitization and degradation (4). Whereas, CD28 induces GRK to phosphorylate the CCR5 receptor, CTLA-4 engagement inactivates GRK2, leading to delaying or preventing phosphorylation of CCR5, and thereby halts desensitization. In addition, CTLA-4-enhanced specific migration might be partly the consequence of integrin-supported chemotaxis (66, 67), but is also mediated by TCR-mediated PI3K-Akt phosphorylation which synergizes with CD28-mediated migration (4). Antigen-attracted T-cells competent for CTLA-4 move specifically to sites of inflammation and easily home to lymph nodes *in vitro* and *in vivo* whereas CTLA-4 incompetent T-cells migrate much less (3, 60). Others have shown that T-cells poorly exit an IFN-treated peritoneal cavity, when before antigen recognition by T-cells anti-CTLA-4 antibodies and anti-hamster antibodies were applied (24). T-cells under this treatment did not move and therefore it is unclear whether the antibody-treatment blocked or crosslinked CTLA-4 and to which degree CTLA-4 operated in trans or without CD28 ligation (4).

Anti-CTLA-4 interference with the interaction between T-cells and DCs (1) laid a precedent for the follow-on finding that PD-1 blockade has similar effects in disrupting T-cell bindings to other cells (5, 68). Antibodies to PD-1 also limit contact times of anergic T-cells (5) and CD8 T-cells (68). In the latter study, PD-L1 was found to localize to the central supramolecular activation cluster, to decrease antiviral CD8 T-cell motility, and promote stable immunological synapse formation. Antibodies to PD-1-PD-L1 restored CD8 T-cell motility in the presence of high viral loads (68).

In this model, anti-PD-1 blockade has shared and distinct properties relative to CTLA-4 blockade. PD-L1 ligation of PD-1 appears to enforce adhesion that is released by anti-PD-1 blockade. PD-1 associated SHP-2 does not appear to negatively regulate adhesion. It is likely that CTLA-4 binding to CD80/86 might also promote adhesion and it blockade might release the T-cell from binding to another cell. However, in addition to this event, anti-CTLA-4 also promotes motility (1, 69). CTLA-4 expressing T-cells simply failed to undergo motility arrest *in vivo* in the presence of antigen, without the need for antibody blockade (1, 50). Antibody blockade of receptor binding to ligand and the induction of motility are therefore likely to cooperate in disrupting T-cell binding to other cells. In the presence of blocking antibody, the natural expression of CTLA-4 might limit contact of T-cells, while the additional blockade with anti-CTLA-4 ensures the complete release of the weakly adhesive T-cells. In both instances, anti-CTLA-4 and PD-1 limit T-cell binding to DCs during antigen presentation, thereby reducing the efficacy of TCR signaling and raising the threshold needed for the activation of T-cells. This is further complicated by the observation that T-cells from CTLA-4 deficient mice fail to stop in response to anti-CD3 ligation (50). It is unclear whether this feature is due to chronic stimulation that might over-ride the stop signal over time. Overall, the current data indicates that CTLA-4 and PD-1 alters the interaction of T-cells with other cells, including antigen-presenting cells, and consequently, alters the overall motility and migration of T-cells. The exact nature of the regulatory effect may vary depending on the nature of the T-cell, whether CTLA-4 ligation occurs, as well as, the inflammatory conditions in the lymphoid microenvironment.

## CTLA-4 and PD-1 blockade in tumor models

**Figure 3:**
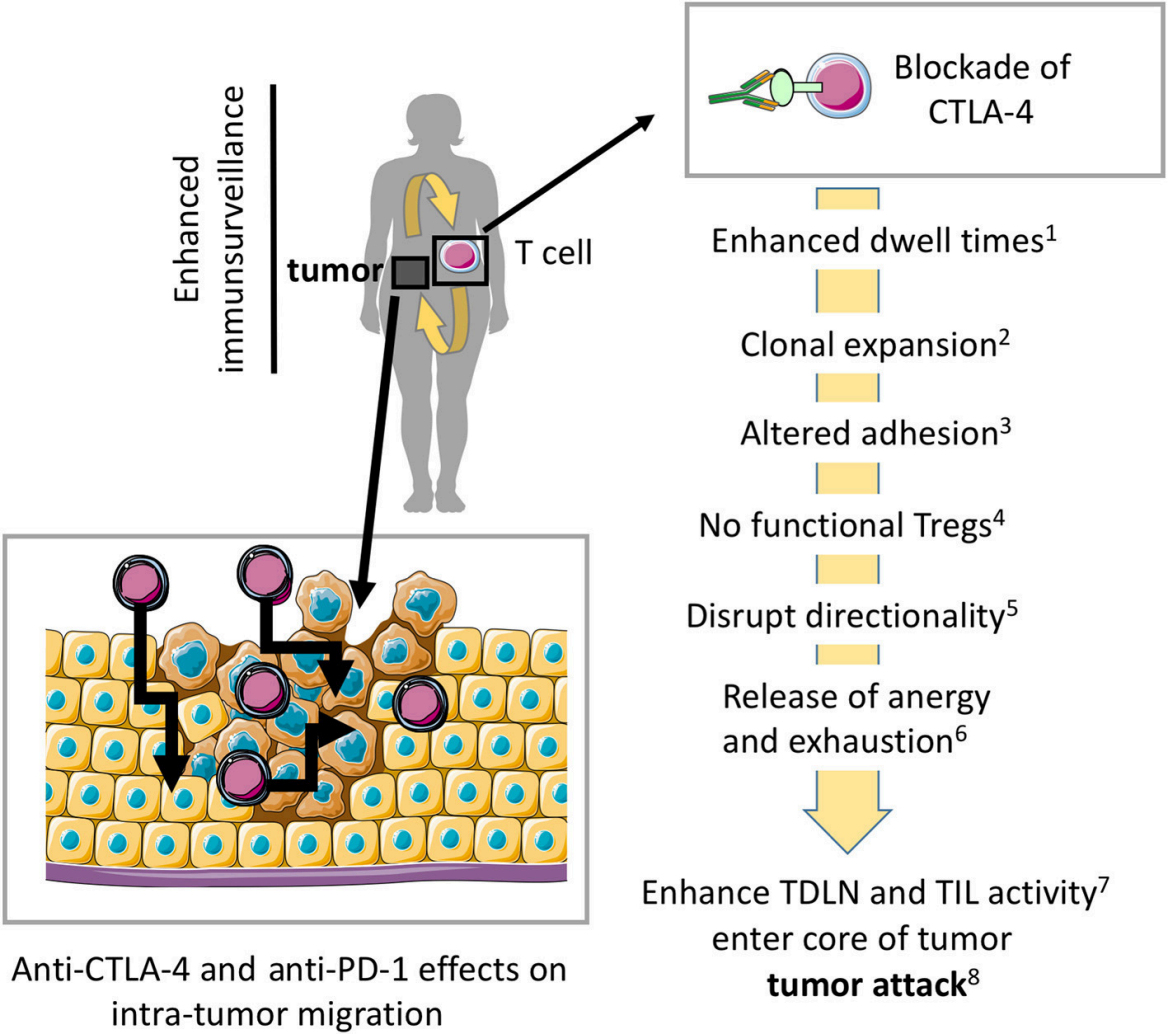
Model where blockade of CTLA-4 and PD-1 enhances migration into tumors and within tumors for more effective tumor rejection. Preventing CTLA-4 engagement, i.e., using anti-CTLA-4-antibodies *in vivo* modulates the entry and migration of T-cells within tumors for more effective tumor elimination. Anti-CTLA-4 and anti-PD-1 effects on antibodies may also modulate T-cell movement within the tumor mass. (1) (5, 43), (2) (28–30, 74), (3) (49), (4) (35, 45), (5) (3, 4, 32), (6) (65, 69), (7) (10, 31, 32), (8) (25, 26, 37, 38). The scheme was drawn using pictures from Servier Medical Art.

A prediction from this work has been that CTLA-4 plays a similar role for T-cell entry and movement in tumors. Many tumors express neo-antigens that can be recognized by resident and peripheral T-cells. This aspect might contribute to the synergy seen between anti-CTLA-4 check-point blockade and other modalities of immune intervention (34, 70–73) (Figure 3). As mentioned, CTLA-4 limits dwell times with DCs and potential other tissues (1, 2) and the *in vitro* and *in vivo* migration of T-cells is enhanced by CTLA-4 (3, 4). In the presence of antibody blockade, T-cells accumulate in the blood and remain circulating in the body (3). Due to angiogenesis, the enhanced presence of circulating T-cells in the blood may provide an advantage in facilitating tumor access (75). In particular, as vessels at the tumor side are highly branched, irregular, and show a discontinued blood flow (75).

Furthermore, the tumor generates a local immune privileged microenvironment where access by T-cells is limited since integrin-mediated extravasation from blood stream is made difficult as ligands are downregulated at the barrier (75). Some tumors may even grow in immune privileged sides such as the central nervous system. Of note, immune privilege is an active process involving induction of inhibitory mechanisms such as the instructed upregulation of CTLA-4 on T-cells, which can accumulate at the border of the privileged side (76, 77). In addition, T-cells in the tumor microenvironment express CTLA-4 so that blockade releases this localization which enables them to even enter immune privileged microenvironments (32). Therefore, under CTLA-4 blockade using specific antibodies, tumors can be reached by migrating T-cells. However, enhanced motility and migration may also explain immune-related adverse events reported under therapy.

As mentioned, anti-CTLA-4 in tumor models has shown to increase T-cell movement in the tumor (61, 69). In murine breast cancer models, CTLA4 blockade using specific antibodies increased the motility of tumor infiltrating lymphocytes (TILs) in the tumor cavity *in vivo* (69). The expression of NKG2D then offset this effect by enhancing TILs arrest. In some manner, this combination of anti-CTLA-4 effects on motility combined with stabilization as mediated by NKG2D enhanced tumor eradication. In general, anti-CTLA-4 check-point blockade has been associated with greater tumor entry, although the exact mechanism for this increase in tumor entry has yet to be determined (51). Similarly, in allo-graft models, anti-CTLA-4 blockade increased motility of CD4 effector and Treg cells, it may decrease the motility of CD8 effector T-cells (10). The explanation for these different effects is unclear but may relate to kinetics of CTLA-4 expression on subpopulations and thus, whether it is expressed under anti-CTLA-4 treatment at the cell surface of T-cell helpers and/or CD8 T-cell attackers (47, 74, 78). As CTLA-4 has a much higher affinity to CD80 and CD86 than CD28, the outcome will also be influenced by CD80/86 expression and subsequent ligation in the TDLN and tumor sites (65, 69).

CTLA-4 and PD-1 may have similar effects on T-cell reactivity against tumors; however, the differences in their mode of action may also suggest differences. For example, the more restricted ability of anti-PD-1 to block PD-1 binding to PD-L1 may suggest a more restricted role for T-cells already localized in tumors. Indeed, anti-PD-1 therapy has been reported to have fairly minor effects in promoting an increase in numbers of TILs in tumors such as melanoma. It predominate function on CD8 T-cells may also lead to a restricted effect on this subset. This may operate in conjunction with the effects of anti-PD-1 in restoring functionality to exhausted T-cells (79). By contrast, the combined effects of blockade and direct enhanced motility may be expected to lead to an increase in the migration of T-cells into and within tumors. At the same time, its effect on CD4 and CD8 T-cells might imply a more generalized role on these two major subsets within the T-cell population. Taken together, under CTLA-4 blockade, immune surveillance may be enhanced to sites where T-cells have restricted for tumor entry such as in peri-tumor sites where T-cells can be paralyzed. The synergy of combinational therapy such as CTLA-4 and PD-1 blockade could be due to enhanced motility and a reversal of T-cell exhaustion on different T-cells and in different microenvironments.

## Conclusion

Although CTLA-4 impinges on many features of T-cell biology, its effect on tissue and tumor infiltration will be the subject of exciting future work. Antibodies to CTLA-4 may act to facilitate tumor entry and alter the movement in tumors, rates of egress. Further studies will elicit and exploit this feature to facilitate tumor entry for more effective tumor eradication.

## Author contributions

MB-W and CR contributed equally to the writing of the manuscript.

### Conflict of interest statement

The authors declare that the research was conducted in the absence of any commercial or financial relationships that could be construed as a potential conflict of interest.
